# The potential therapeutic role of melatonin in organ fibrosis: a comprehensive review

**DOI:** 10.3389/fmed.2024.1502368

**Published:** 2024-12-13

**Authors:** Wei Huang, Juan Zheng, Ming Wang, Ling-Yao Du, Lang Bai, Hong Tang

**Affiliations:** ^1^Center of Infectious Diseases, West China Hospital of Sichuan University, Chengdu, China; ^2^Laboratory of Infectious and Liver Diseases, Institute of Infectious Diseases, West China Hospital of Sichuan University, Chengdu, China

**Keywords:** melatonin, fibrosis, therapeutics, protective effects, mechanisms

## Abstract

Organ fibrosis is a pathological process characterized by the inability of normal tissue cells to regenerate sufficiently to meet the dynamic repair demands of chronic injury, resulting in excessive extracellular matrix deposition and ultimately leading to organ dysfunction. Despite the increasing depth of research in the field of organ fibrosis and a more comprehensive understanding of its pathogenesis, effective treatments for fibrosis-related diseases are still lacking. Melatonin, a neuroendocrine hormone synthesized by the pineal gland, plays a crucial role in regulating biological rhythms, sleep, and antioxidant defenses. Recent studies have shown that melatonin may have potential in inhibiting organ fibrosis, possibly due to its functions in anti-oxidative stress, anti-inflammation, remodeling the extracellular matrix (ECM), inhibiting epithelial-mesenchymal transition (EMT), and regulating apoptosis, thereby alleviating fibrosis. This review aims to explore the therapeutic potential of melatonin in fibrosis-related human diseases using findings from various *in vivo* and *in vitro* studies. These discoveries should provide important insights for the further development of new drugs to treat fibrosis.

## 1 Introduction

Organ fibrosis is the ultimate outcome of various chronic diseases, characterized by the deposition of extracellular matrix (ECM) leading to scar tissue formation as the primary pathological change (1). Continuous progression can result in structural damage and loss of function in organs, ultimately leading to death, as seen in end-stage liver, kidney, lung, and heart diseases (2). Organ fibrosis significantly impacts global morbidity and mortality rates. In developed countries, nearly 45% of human deaths are associated with fibrosis-related diseases, and this proportion may be even higher in developing countries (3). Despite ongoing research into the occurrence and development of fibrosis, current methods for anti-fibrotic treatment are limited and often ineffective, making it crucial to search for effective therapeutic approaches.

Melatonin (N-Acetyl-5-methoxytryptamine) is a methoxylated indole produced in peripheral tissues such as the retina, gastrointestinal tract and bone marrow and primarily synthesized and secreted by the pineal gland under normal light–dark conditions during the night in vertebrates (4). Melatonin was first isolated from the bovine pineal gland by Lerner et al. (5). Melatonin synthesis begins with the hydroxylation of tryptophan to form 5-hydroxytryptophan, followed by decarboxylation to produce 5-hydroxytryptamine (5-HT). This is then acetylated to form N-acetyl-5-hydroxytryptamine, which is finally methylated to produce N-acetyl-5-methoxytryptamine (6). The production of melatonin is typically influenced by various factors, and it is currently understood that the secretion rhythm of melatonin relies on an intrinsic circadian structure triggered by light signals received by the retina. This rhythm originates from the suprachiasmatic nucleus (SCN), where light exposure transmits neural impulses to the SCN via the retinohypothalamic tract, synchronizing the activity of the SCN and the nocturnal secretion rhythm of melatonin with the 24-h light/dark cycle. Norepinephrine (NE) serves as a mediator in the effect of light on melatonin synthesis and secretion. Light can inhibit the release of NE through specific pathways, whereas in darkness, sympathetic neuronal activity within the pineal gland significantly increases, resulting in higher NE release. NE binds to receptors on the pineal gland cell membrane, promoting the entry of tryptophan into the cells. Once NE binds to its receptors, cyclic adenosine monophosphate (cAMP) facilitates the synthesis of serotonin and melatonin (7). Additionally, light is an important external factor influencing melatonin secretion. Various non-environmental factors, such as sleep quality and duration, hunger, and physical activity, may also affect melatonin secretion (8–10).

After melatonin is synthesized, it is immediately released into the cerebrospinal fluid and blood, distributing throughout the body via systemic circulation. Its plasma concentration exhibits a distinct circadian rhythm, being higher at night than during the day (11). Based on the affinity for binding sites, melatonin receptors can be classified into three subtypes: melatonin receptor 1A (MT1) (12), melatonin receptor 1B (MT2) (13), and melatonin receptor 1C (MT3) (14). As members of the G protein-coupled receptor superfamily, MT1 and MT2 are both primarily located in the brain and other extra-pineal tissues, including the liver, skeletal muscle, and retina. MT3 is found in the liver, kidneys, heart, adipose tissue, and brain (15). By binding to different melatonin receptors, melatonin rapidly activates various signal transduction cascades, thereby exerting its biological effects in the body. Melatonin is widely recognized as a potent antioxidant and is also involved in mediating various biological effects such as anti-inflammatory responses, circadian rhythms, cellular metabolism, and immune regulation (16). Recent research suggests that exogenous melatonin supplementation can alleviate organ fibrosis, including liver fibrosis, lung fibrosis, heart fibrosis, and kidney fibrosis, indicating that melatonin may be a potential anti-fibrotic agent.

This review begins by outlining the process of liver fibrosis development. It then summarizes the possible mechanisms by which melatonin inhibits fibrosis development. Finally, it discusses the roles melatonin may play in different organ fibrosis scenarios. It is hoped that this article will provide a new perspective for subsequent experimental research and offer more theoretical support for the clinical recommendation and widespread application of melatonin.

## 2 Pathogenesis of organ fibrosis

**Figure 1:**
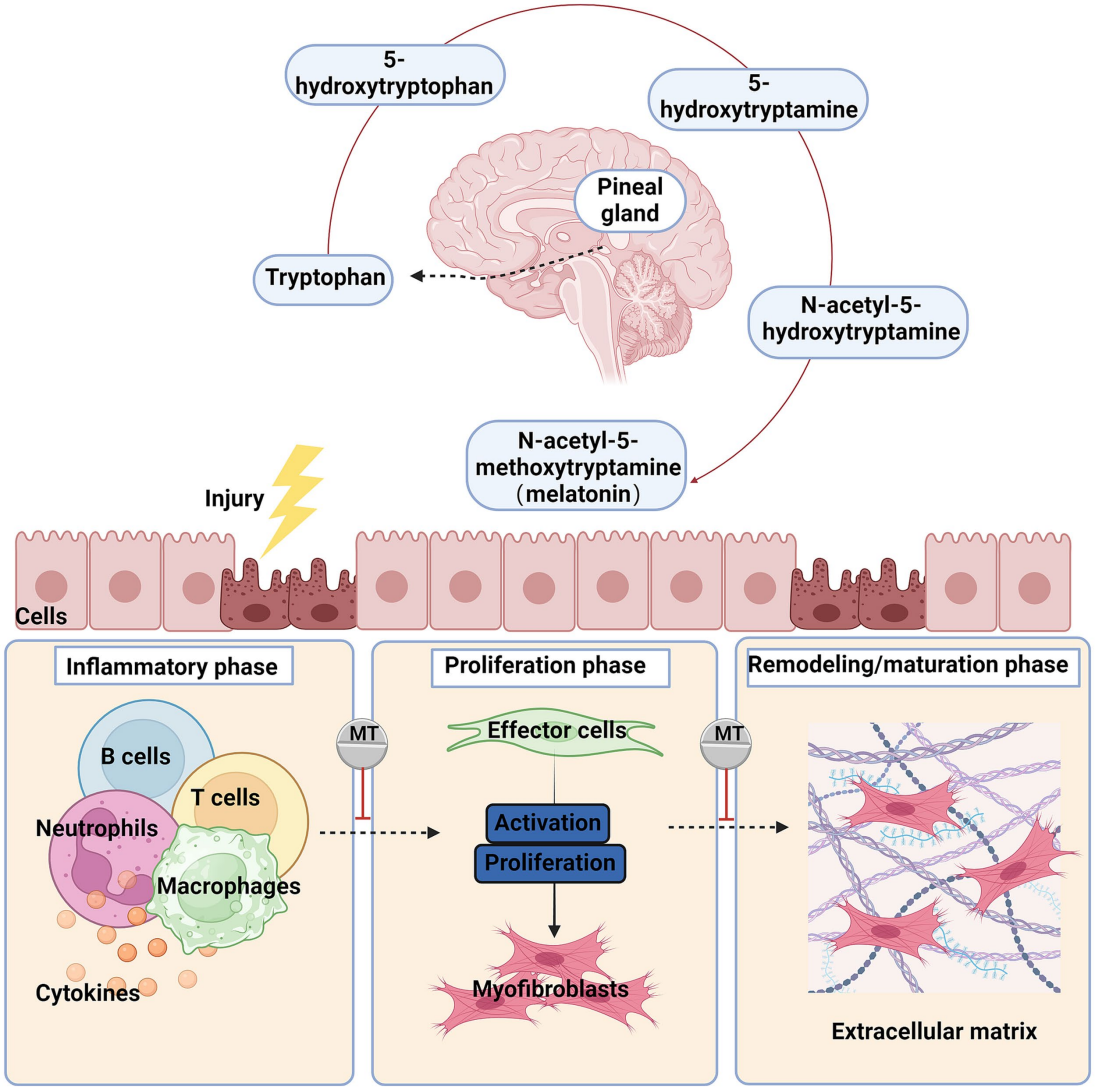
The primary synthesis process and anti-fibrotic effects of melatonin after organ injury, the damaged cells secrete chemokines and cytokines, recruiting and activating inflammatory cells such as macrophages, monocytes, and lymphocytes. The signals released by these cells activate fibroblasts, which then transform into myofibroblasts and synthesize extracellular matrix (ECM). Chronic injury and inflammation lead to persistent myofibroblast activation, excessive ECM deposition, and ultimately fibrosis. Red blunted line: an inhibitory effect of melatonin.

Typically, when tissue is injured, wound healing undergoes three main phases: the inflammatory phase, the proliferative phase, and the remodeling/maturation phase (2, 17). These phases serve different functions and overlap in time (18) (Figure 1).

Under normal circumstances, after an injury, the formation of a platelet plug and provisional ECM stops the bleeding, accompanied by an increased threshold of the inflammatory response and recruitment of immune cells. This process initiates the first phase of healing, the inflammatory phase. During this phase, a large number of neutrophils and macrophages infiltrate the tissue to combat potential infection and clear debris (19). In the inflammatory phase, damaged endothelial/epithelial cells and myofibroblasts produce aberrant matrix metalloproteinases (MMPs), disrupting the basement membrane of local tissues and releasing various cytokines and chemokines, thereby recruiting and activating more immune cells, including neutrophils, macrophages, T lymphocytes, and B lymphocytes (3, 20).

Activated leukocytes release mediators such as pro-inflammatory, vasoactive, and pro-fibrotic effectors, inducing precursor cells to differentiate into myofibroblasts. Myofibroblasts rapidly produce large amounts of ECM to maintain the integrity of damaged tissue during the repair process and promote cell proliferation to form granulation tissue. Myofibroblasts are the primary effector cells in fibrosis (21).These cells can secrete large amounts of ECM proteins (mainly collagen I, collagen III, and fibronectin), increase the production of tissue inhibitors of metalloproteinases, and express alpha smooth muscle actin (*α*-SMA), which confers contractility (22, 23). The sources of myofibroblasts may vary across different tissues. Currently, it is believed that the activation of resident fibroblasts in the tissue is the main source of myofibroblasts (24). Under certain conditions, epithelial and endothelial cells also can differentiate into myofibroblasts through epithelial/endothelial-mesenchymal transition (EMT/EndoMT) (25–27), and mesothelial cells can differentiate into myofibroblasts through mesothelial-mesenchymal transition (28, 29).

In the final tissue remodeling/maturation phase, activated myofibroblasts cause wound contraction; dynamic ECM degradation and remodeling restore the parenchymal tissue structure. However, persistent inflammation, necrotic cells, ongoing fibroblast activation, and excessive ECM deposition lead to abnormal tissue reconstruction, resulting in organ fibrosis.

To date, various mediators have been identified that can activate fibroblasts and promote the occurrence and progression of fibrosis, including transforming growth factor-β (TGF-β) (30), interleukins (IL-1β, IL-6, IL-13, IL-33, IL-11, IL-17, etc.), tumor necrosis factor-*α* (TNF-α), platelet-derived growth factor (PDGF), connective tissue growth factor (CTGF) (31), angiotensin-II (Ang-II) (32), heat shock protein (33), endothelin-1 (34), integrins (35), reactive oxygen species (ROS), and hypoxia. TGF-β is a cytokine with diverse physiological functions, playing a key role in regulating cell proliferation, differentiation, apoptosis, and immune responses (36). Currently, three isoforms of TGF-β are known: TGF-β1, TGF-β2, and TGF-β3. Among these, TGF-β1 is the most abundant isoform in humans, widely expressed in most cell types, with platelets serving as a significant source (37). Although these three isoforms share similar bioactive regions and can bind to the same type I and type II TGF-β receptor complexes, TGF-β is widely recognized as the most potent pro-fibrotic factor, primarily acting through the Smad (Smad-2, −3, −4, etc.) signaling pathway to promote fibrosis development (38, 39). In addition, TGF-β can also facilitate fibrosis through multiple non-Smad-dependent pathways, such as those involving c-Jun N-terminal kinase (JNK), p38 mitogen-activated protein kinase (p38 MAPK), extracellular signal-regulated kinase (ERK1/2), and phosphatidylinositol 3-kinase/Akt (PI3K/Akt) or Rho-like GTPases (30, 40). During TGF-β signaling, activated Smad complexes can induce the expression of various transcription factors in the nucleus, thereby promoting the activation of fibroblasts. Research indicates that the Smad pathway can induce the expression of the JunD transcription factor, and knocking out this gene results in a reduction in the number of myofibroblasts and decreased collagen release (41). Additionally, the Smad pathway interacts with the Snail family of transcription factors (42) and zinc finger E-box-binding homeobox (ZEB) transcription factor family to enhance myofibroblast differentiation through EMT (43). IL-1β-treated fibroblasts differentiate into myofibroblasts, leading to increased ECM deposition. IL-13 promotes liver fibrosis formation by directly inducing the expression of liver fibrosis-related genes such as collagen and CTGF. TNF-*α* is a critical inflammatory signal molecule in fibrosis, mainly secreted by macrophages. By binding to specific receptors, TNF-α initiates signal cascades, activating nuclear factor-kappa B (NF-κB) to regulate inflammatory responses, and triggers cell activation, differentiation, cytokine production, and apoptosis, thus contributing to fibrosis progression. NF-κB is an inducible transcription factor family responsible for regulating the initiation and progression of inflammatory responses. The activation of NF-κB occurs primarily through two pathways: the canonical and the non-canonical pathways. The canonical pathway is activated when signals such as pro-inflammatory cytokines or pathogen-associated molecular patterns activate cell surface receptors, including pattern recognition receptors, Toll-like receptors (TLR), and T cell receptors. In contrast, the non-classical signaling pathway is primarily activated by TNF and its corresponding TNF receptors (44). Studies have shown that during the NF-κB signaling process, its inhibitors can suppress fibroblast activity in a concentration-dependent manner (45).

## 3 The mechanisms of melatonin alleviating fibrosis

**Figure 2:**
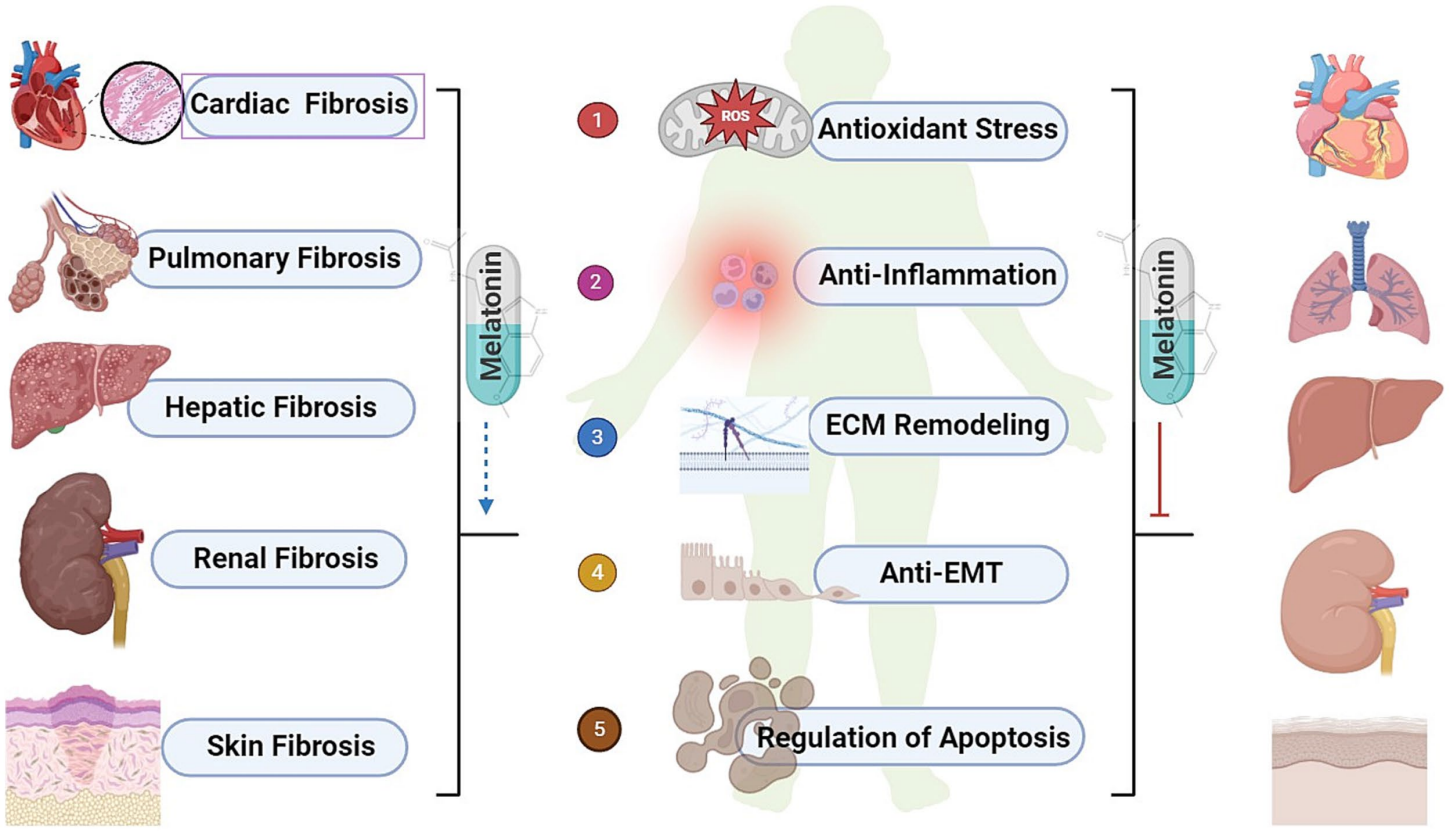
The biological effects of melatonin and common organ fibrosis. ROS, reactive oxygen species; ECM, extracellular matrix; EMT, epithelial-mesenchymal transition.

Melatonin was initially discovered and named for its ability to lighten the skin of amphibians (5, 46). As research progressed, melatonin was identified as a lipid-soluble hormone that can act on almost every cell in the organism, crossing all biological barriers. Its primary function is to regulate and reset circadian rhythms (47), but it also has anti-cancer (48), immune-modulating (49) and many other functions. Melatonin exerts its anti-fibrotic effects, and the mechanisms may involve reducing oxidative stress, inhibiting inflammatory responses, ECM remodeling, suppressing EMT, and preventing apoptosis (Figure 2).

### 3.1 Antioxidant stress

Oxidative stress arises from cells’ inefficient utilization of molecular oxygen (50). ROS include superoxide anion radicals, hydroxyl radicals, hydrogen peroxide, and singlet oxygen, which are byproducts of cellular respiration and other metabolic processes (51). Additionally, there are highly destructive nitrogen-based substances such as nitric oxide, especially peroxynitrite anion (52). Oxidative stress is considered a major cause of fibrosis as it damages the structures of cellular macromolecules like DNA, proteins, and lipids, ultimately leading to cell damage and promoting fibrosis (53).Studies have shown that melatonin can enhance mitochondrial adenosine triphosphate synthesis and reduce the production of reactive oxygen species (54). Furthermore, melatonin not only directly interacts with various ROS, reactive nitrogen species, and organic radicals to exert a direct scavenging effect, but also upregulates the activities of antioxidant enzymes such as superoxide dismutase (SOD), catalase (CAT), glutathione peroxidase (GPx), glutathione, (GSH), glutathione reductase (GRd), and glucose-6-phosphate dehydrogenase (52, 55). Additionally, melatonin downregulates the activities of pro-oxidant enzymes like nitric oxide synthase (NOS) and lipoxygenase, thereby exerting an indirect antioxidant effect (52). Furthermore, melatonin, similar to metallothioneins, can bind to heavy metals such as aluminum, cadmium, copper, iron, lead, and zinc, thus mitigating oxidative stress (56). As a lipid-soluble free radical scavenger, melatonin can easily cross the blood–brain barrier, replacing or supplementing metallothioneins in the brain as the primary binding agents for transition metals (57).

### 3.2 Anti-inflammation

Recruited inflammatory cells and damaged epithelial cells eventually transform into fibrogenic effector cells or induce the activation of precursor cells, which is a crucial component of the fibrosis process. Recent studies have found that melatonin can inhibit the infiltration of inflammatory cells by reducing myeloperoxidase activity (58, 59). Melatonin effectively lowers the levels of pro-inflammatory factors and fibrosis markers, demonstrating anti-inflammatory activity in various diseases. In human blood cells, melatonin can reduce lipopolysaccharide (LPS)-induced TNF-*α* levels (60, 61). In patients with osteoarthritis, researchers observed that melatonin effectively inhibits the production of pro-inflammatory cytokines during inflammation by suppressing the Erk and PI3K/Akt signaling pathways, significantly reducing the expression of TNF-α, IL-8, and vascular endothelial growth factor in synovial fibroblasts (62). The NF-κB transcription factor family is considered a central mediator in the inflammatory process (63), and studies have shown that melatonin can inhibit NF-κB signaling and activate the antioxidant regulators nuclear erythroid 2-related factor 2 (Nrf2) (63, 64). Furthermore, melatonin exerts its anti-inflammatory effects by inhibiting the expression of the inflammasome NLR family pyrin domain containing 3 (NLRP3) and the activation of NF-κB while upregulating the expression of the transcription factor Nrf2 (65). Melatonin also significantly reduces the expression levels of nitric oxide and malondialdehyde (MDA), which are closely associated with inflammation (59, 66).

### 3.3 ECM remodeling

Under physiological conditions, the ECM is a highly dynamic structure present in all tissues, constantly undergoing balanced remodeling. This remodeling process is primarily mediated by specific enzymes, particularly MMPs (67). When tissue is damaged, the synthesis and degradation of ECM become progressively imbalanced, eventually leading to ECM deposition and, consequently, tissue fibrosis (68). During ECM deposition, a large amount of MMPs is activated, breaking down the cells and their surrounding basement membrane matrix, allowing leukocytes to migrate into the tissue (69). Additionally, MMPs influence the secretion of various chemokines and cytokines, creating a strong chemokine gradient that leads to the recruitment of inflammatory cells to the site of injury (70, 71). Melatonin has a regulatory effect on MMP gene expression and catalytic activity (72). MMP-9 is a zinc metalloproteinase that, as a matrix protein, is released from intracellular stores and becomes active extracellularly (73, 74). Studies have found that in mice with liver fibrosis treated with melatonin, MMP-9 activity is reduced and Nrf2 expression is increased (75). In gastric adenocarcinoma cell lines, melatonin inhibits both the induction and catalytic activity of MMP-9 (73). Collagen and glycosaminoglycan (GAG) are major components of the ECM, and research has shown that melatonin can effectively reduce the amounts of collagen (76–78) and GAG (79), thereby alleviating tissue fibrosis to some extent.

### 3.4 Anti-EMT

EMT is an important process in normal embryonic development and tissue repair, during which differentiated epithelial cells undergo phenotypic transformation to acquire a mesenchymal phenotype (80). This is a reversible biological process. The EMT process involves the induction and regulation of multiple signal transduction pathways, primarily including TGF-β, NF-κB, Wnt, Notch and et. pathways (67). These pathways converge on the EMT-related transcription factor families, triggering extensive cellular transcriptional reprogramming. The main EMT-related transcription factors include members of the SNAIL, ZEB, TWIST, and PRRX families, as well as various EMT effectors (81). In the context of persistent chronic injury, the role of EMT shifts from promoting tissue repair to contributing to tissue degeneration, leading to irreversible dedifferentiation and cell cycle arrest (82). Moreover, EMT can enhance the secretion of TGF-β and inflammatory cytokines by damaged epithelial cells, which, respectively, promote the differentiation of fibroblasts into myofibroblasts and trigger immune inflammation, ultimately resulting in repair failure and tissue fibrosis (83, 84). Research indicates that melatonin may inhibit the activity of the Notch signaling pathway, thereby blocking the migration, invasion, and EMT of normal and endometriosis epithelial cells induced by 17β-estradiol (85). Research indicates that melatonin can inhibit the secretion of IL-1β, IL-6, and TGF-β induced by LPS both *in vivo* and *in vitro* (86). Additionally, melatonin can reverse LPS-induced EMT in peritoneal mesothelial cells by inhibiting the TLR4/ JNK and TLR4/NF-κB-Snail signaling pathways (87).

### 3.5 Regulation of apoptosis

Apoptosis is an orderly process of programmed cell death controlled by genes, involving the activation, expression, and regulation of genes (88). It is a proactive response of cells to adapt to their living environment. The regulation of apoptosis mainly occurs through the intrinsic mitochondrial pathway and the extrinsic death receptor pathway (89). The intrinsic pathway is initiated by the insertion of Bax/Bak into the mitochondrial membrane, followed by the release of cytochrome c (Cyt C) (90). Cyt C then combines with Apaf-1 and procaspase-9 to form the apoptosome, which subsequently activates caspase-3, initiating the apoptotic cascade (91). The extrinsic pathway is triggered by external stimuli or ligand molecules, primarily involving death receptors (92). Research indicates that melatonin can reduce cisplatin-induced apoptosis in renal tubular cells by inhibiting the activation of caspase-3 (93). In thymocytes induced by glucocorticoids, melatonin exerts an anti-apoptotic effect by regulating the levels of Bax protein (94). During fibrosis, the abnormal proliferation of fibrotic effector cells and their resistance to apoptosis are key factors. Interestingly, the regulation of apoptosis by melatonin depends on the cell type. In human hypertrophic scar tissue, melatonin inhibits fibroblast proliferation and induces apoptosis by regulating the gene expression of cyclin E, p53, and Fas (95). The alleviation of fibrosis by melatonin may be related to the reduction of fibroblast migration through decreased chloride channel activity mediated by protein kinase C (96).

## 4 Protective effects of melatonin in fibrotic diseases

Numerous studies have shown that melatonin provides comprehensive protection for various organs and tissues, including the heart, lungs, liver, and kidneys, by preventing fibrosis and aiding in the repair of damage to these organs and tissues (Table 1).

**Table 1:** Summary of anti-fibrotic effects and underlying mechanisms of melatonin.

Fibrotic disease	Model	Animal/cell type	Effect and mechanism	References
Cardiac fibrosis	High-fat diet and PM2.5	Male ApoE^−/−^ mice	Reversing phenotypic modulation of cardiac fibroblasts into myofibroblasts; ↓mitochondrial ROS generation and oxidative injury; regulating SIRT3-mediated SOD2 deacetylation	Jiang et al. (103)
	Ang-II and PM2.5	Embryonic heart fibroblast cell		
	Streptozotocin	Male KM mice	↓Collagen production; ↓lncR-MALAT1/miR-141-mediated NLRP3 inflammasome activation and TGF-β1/Smads signaling	Che et al. (104)
	High-glucose	Cardiac fibroblast		
	Abdominal aortic constriction	Male SD rat	↓Cross-sectional area of myocardial fibers and collagen deposition; ↓HDAC1-4 and HDAC6 expressions	Wu et al. (106)
	High-fat/Streptozotocin and MI surgery	Male C57BL/6 J mice	↓Cardiomyocyte cross-sectional area and interstitial fibrotic area; ↓p53, caspase-3, and Bax levels; ↑Bcl-2 levels; ↓the JNK/p53-mediated apoptotic pathway	Lu et al. (107)
	High fat/High glucose and hypoxia	Cardiomyoblast		
	High-glucose and MI/R surgery	Male SD rat	↓Infarct size, ↓TUNEL-positive myocardial cells and oxidative stress; ↑Notch1/Hes1/Akt signaling; rescued intracellular Trx system	Yu et al. (109)
	High-glucose	Cardiomyoblast		
Pulmonary fibrosis	Bleomycin	Male Wistar rat	↓Collagen accumulation; attenuating airway dysfunction; ↓COX-2 expression	Karimfar et al. (78)
	Bleomycin	Male Wistar rat	↓HYP content; ↑the CAT activity	Yildirim et al. (112)
	LPS	Alveolar epithelial cell	↑E-cadherin expression; ↓α-SMA expression; activating the PI3K/Akt signaling pathway;↑GSK-3β phosphorylation and Nrf2 protein	Ding et al. (114)
	TGF-β1	Alveolar epithelial cell	↓Vimentin and N-cadherin expression; ↑E-cadherin expression; ↓the Wnt/β-catenin and Smad signaling pathways	Yu et al. (117)
Hepatic fibrosis	PM2.5	Female C57BL/6 J mice	↑MDA and ROS levels; ↓GSH levels; ↓inflammatory factors levels; activating Nrf2	Zhu et al. (122)
		Hepatic stellate cell		
	CCl4	Male SD rat	↓ALT and AST levels;↓the serum LN, HA and HYP levels; ↑GPx levels; ↓MDA levels	Hong et al. (123)
	CCl4	Male C57BL/6 J mice	↓Collagen I and III, TGF-β, PDGF, CTGF, doublecortin-like kinase, and p-Smad3 expression; ↓MMP-9 activity; ↑Nrf2 expression	Crespo et al. (75)
	Bile-duct ligation	Male Wistar rat	↑SOD and GSH levels; ↓MDA levels; ↓TUNEL-positive cells; ↓*α*-SMA expression	Aktas et al. (124)
Renal fibrosis	Cadmium chloride	Male C57BL/6 mice	↑SOD, GSH, and CAT activities;↓MDA levels; ↓TNF-α and iNOS expression; ↓caspase-3 and Bax expression; ↑Bcl-2 expression; ↓collagen deposition and fibrosis	Yang et al. (128)
	Lactacystin	Male Wistar rat	↓HYP levels; ↓the ratio and total amount of collagen I and III	Repova et al. (129)
	TGF-β1	Renal interstitial fibroblast	↓The proliferation and activation of renal interstitial fibroblast;↓ROS and MDA levels; ↑GSH/oxidized GSH ratio; ↓Smad and non-Smad signaling cascades	Kim et al. (130)
Skin fibrosis		Skin fibroblast	↓the migration and contractility of HSF; ↓ collagen and α-SMA production; ↓ PI3K/Akt/mTOR signaling	Dong et al. (134)
	Wound on ventral surface of ear	Male New Zealand white rabbit		
		Keloid fibroblast	↑c-PARP, c-caspase3 and c-caspase9 expression; ↓cell proliferation, migration and invasion, contractile capability and collagen production; ↓the Erk and Smad pathways	Huang et al. (135)

PM2.5, fine particulate matter; Ang-II, angiotensin-II; ROS, reactive oxygen species; SIRT3, sirtuin3; SOD2, superoxide dismutase 2; NLRP3, NLR family pyrin domain containing 3; TGF-β1, transforming growth factor-β1; HDAC, histone deacetylases; MI, myocardial infarction; JNK, c-Jun N-terminal kinase; MI/R, myocardial ischemia–reperfusion; Trx, thioredoxin; COX2, cyclooxygenase 2; HYP, hydroxyproline; CAT, catalase; LPS, lipopolysaccharide; α-SMA, alpha smooth muscle actin; PI3K/Akt, phosphatidylinositol 3-kinase/Akt; GSK-3β, glycogen synthase kinase 3β; Nrf2, nuclear erythroid 2-related factor 2; ALT, alanine aminotransferase; AST, aspartate aminotransferase; CCl4, carbon tetrachloride; LN, laminin; HA, hyaluronic acid; GPx, glutathione peroxidase; MDA, malondialdehyde; PDGF, platelet-derived growth factor; CTGF, connective tissue growth factor; p-Smd3, phosphorylated Smad3; MMP-9, matrix metalloproteinase 9; SOD, superoxide dismutase; GSH, glutathione; TNF-α, tumor necrosis factor-α; iNOS, inducible nitric oxide synthase; HSFs, hypertrophic scar fibroblasts.

### 4.1 Melatonin and cardiac fibrosis

Cardiac fibrosis is the ultimate pathological outcome of various cardiovascular diseases, commonly seen in myocardial infarction (MI), hypertension, myocarditis, cardiomyopathy, arrhythmias, diabetes and radiation exposure (97). Its main pathological features include excessive proliferation of cardiac fibroblasts and ECM protein deposition, leading to progressive diastolic and systolic dysfunction, which eventually results in chronic heart failure, and sudden cardiac arrest (98, 99). Numerous studies have shown that melatonin affects the cardiovascular system. In cardiovascular diseases, levels of melatonin are found to be reduced (100–102). Melatonin therapy can effectively alleviate fibrosis following these diseases. Melatonin can mitigate cardiac dysfunction and fibrosis induced by fine particulate matter (PM2.5) in mice by inhibiting the phenotypic transformation of cardiac fibroblasts into myofibroblasts. This mechanism may involve the suppression of mitochondrial oxidative damage and regulation of sirtuins 3 (SIRT3)-mediated SOD2 deacetylation (103). MiR-141 is an upstream factor in cardiac fibrosis, involved in regulating NLRP3 and TGF-β1. Melatonin can improve cardiac function and reduce collagen production in diabetic mice by inhibiting the lncR-MALAT1/miR-141 mediated activation of the NLRP3 inflammasome and TGF-β1/Smads signaling pathway (104).Histone deacetylases (HDAC) are involved in several processes related to cardiovascular diseases, including cardiac hypertrophy, remodeling, and fibrosis (105). Melatonin treatment can exert anti-fibrotic effects by inhibiting HDAC expression (106). Additionally, melatonin treatment can alleviate cardiac injury and fibrosis in diabetic mice following MI by inhibiting the JNK/p53-mediated apoptotic pathway. This inhibition reduces the levels of p53, caspase-3, and Bax, while increasing the level of Bcl-2 (107). It is well known that Notch1 plays a significant role in cardiac injury repair (108), and melatonin enhances Notch1/Hes1/Akt signaling in a receptor-dependent manner, rescuing the intracellular thioredoxin (Trx) system, reducing infarct size, cardiomyocyte apoptosis, and oxidative stress, thereby improving myocardial ischemia–reperfusion injury (109).

### 4.2 Melatonin and pulmonary fibrosis

Pulmonary fibrosis is an end-stage lung condition caused by various factors, characterized by fibroblast proliferation and extensive ECM deposition, leading to the destruction of lung tissue structure (110). Pulmonary fibrosis is a major clinical outcome of most chronic respiratory diseases, such as chronic obstructive pulmonary disease (COPD), idiopathic pulmonary fibrosis (IPF), acute respiratory distress syndrome (ARDS), radiation and those caused by environmental exposures [e.g., inorganic mineral dust, carbon tetrachloride (CCl4), and bleomycin]. Numerous studies have shown that melatonin can alleviate pulmonary fibrosis caused by various factors. In bleomycin-induced pulmonary fibrosis, melatonin reduces the infiltration and accumulation of inflammatory cells in the alveolar walls (111), as well as the expression of inflammatory mediators such as cyclooxygenase 2 (COX-2) (78). Additionally, melatonin inhibits the increase in hydroxyproline content and the decrease in CAT activity in lung tissue, preventing fibrosis progression by suppressing protein and lipid peroxidation reactions (112). As a key protective regulator in lung diseases, research has found that melatonin also increases the expression of Apelin 13 and inhibits the production of ROS, thereby restoring mitochondrial function and reducing cell apoptosis and senescence during lung injury (113). Melatonin can also effectively inhibit LPS-induced EMT in human type II alveolar epithelial cells (AECs II) by activating the PI3K/Akt signaling pathway, leading to glycogen synthase kinase 3β (GSK-3β) phosphorylation and Nrf2 protein upregulation (114).Furthermore, the Wnt/β-catenin pathway may be involved in various pathological processes of IPF, such as inducing EMT in damaged AECs II and fibrotic cell migration (115, 116). Melatonin inhibits TGF-β1-induced EMT in alveolar epithelial cells by downregulating the Wnt/β-catenin and Smad signaling pathways (117).

### 4.3 Melatonin and hepatic fibrosis

Hepatic fibrosis is a pathological process resulting from liver damage and inflammatory responses caused by various factors such as chronic viral infection, excessive alcohol consumption, exposure to toxic substances, biliary diseases, autoimmune hepatitis (AIH), and non-alcoholic steatohepatitis (NASH). This leads to the activation of hepatic stellate cells (HSCs) and excessive ECM deposition (118). Continued progression of liver fibrosis results in severe complications such as cirrhosis and liver cancer and commonly needs liver transplantation (119).

Numerous *in vivo* and *in vitro* experiments have demonstrated that melatonin treatment has a significant effect on alleviating liver fibrosis (120).As the key effector cells in the process of liver fibrosis, HSCs can be activated under conditions of inflammation and oxidative stress, leading to excessive proliferation and transdifferentiation into myofibroblasts. These myofibroblasts express large amounts of *α*-SMA and ECM proteins, promoting liver fibrosis (121). Melatonin not only reversed the PM2.5-induced increase in MDA and ROS levels and the decrease in GSH levels by activating Nrf2 but also reduced the expression levels of inflammatory factors such as IL-1β, TNF-α, NF-κB, and NLRP3, thereby improving liver function and inhibiting HSCs activation to exert anti-fibrotic effects (122). Melatonin can increase GPx activity and reduce serum markers of liver fibrosis, including hyaluronic acid (HA), hydroxyproline (HYP), and laminin (LN), thereby alleviating CCl4-induced liver dysfunction and fibrosis in rats (123).Additionally, melatonin reduces the expression of collagen I and III, TGF-β, PDGF, CTGF, doublecortin-like kinase, and phosphorylated Smad3, as well as MMP-9 activity, while increasing Nrf2 expression. By limiting multiple pro-fibrotic gene pathways, melatonin alleviates CCl4-induced liver fibrosis in mice (75). In bile duct-ligated rats, melatonin exerts antioxidant effects by increasing SOD and GSH activities, while also reducing hepatocyte apoptosis, thereby alleviating cholestatic liver injury, bile duct proliferation, and fibrosis (124).

### 4.4 Melatonin and renal fibrosis

Renal fibrosis is caused by damage to intrinsic cells (e.g., glomerular podocytes, endothelial cells, mesangial cells, and proximal tubule epithelial cells) due to various pathogenic factors such as ischemia, infection, obstruction, autoimmune diseases, drug injury, hypertension, and diabetes. This damage progresses to extensive collagen deposition and accumulation, leading to the gradual hardening of the renal parenchyma, scar formation, and eventually the complete loss of kidney function (125). It is estimated that chronic kidney disease (CKD) affects 13% of the global population, imposing a significant economic burden on society (126). Renal fibrosis is a common progressive and irreversible pathological feature of CKD, characterized by glomerulosclerosis, tubular atrophy, chronic interstitial inflammation and fibrosis, and vascular rarefaction (127). Currently, there are no effective treatments for renal fibrosis. Research indicates that melatonin can exert antioxidant effects by increasing the activities of SOD, GSH, and CAT and reducing MDA levels in damaged renal tissue. It also exerts anti-inflammatory effects by decreasing the expression of TNF-*α* and inducible NOS (iNOS), and anti-apoptotic effects by downregulating the expression of caspase-3 and Bax and upregulating the expression of Bcl-2. Consequently, melatonin inhibits the expression of fibrosis-related genes and improves renal damage caused by cadmium exposure in mice (128). Melatonin significantly reduced hydroxyproline levels and the ratio and total amount of collagen I and III in the glomeruli and tubular interstitium in rat with lactoferrin-induced renal fibrosis (129). Additionally, melatonin can inhibit ROS in a receptor-independent manner by suppressing both Smad and non-Smad signaling cascades. This prevents the TGF-β1-induced transdifferentiation of renal interstitial fibroblasts into myofibroblasts, thereby alleviating renal fibrosis (130).

### 4.5 Melatonin and skin fibrosis

Skin fibrosis is characterized by the excessive proliferation of fibroblasts and the deposition of excessive ECM in the dermis, with abnormal cross-linking types, leading to skin hardening and damage (131). Progressive fibrosis mediated by myofibroblasts can invade the skin, causing skin fibrotic diseases, including hypertrophic scar (HS), keloids, localized scleroderma (LS), and systemic sclerosis (SSc), chronic graft-versus-host disease (GVHD) (132). The global impact of skin fibrosis is significant, affecting over 100 million people annually in developed countries (133). Research has found that melatonin enhances autophagy by inhibiting PI3K/Akt/mTOR signaling through the MT2 receptor, thereby inhibiting the migration and contractility of HS fibroblasts (HSFs), as well as the production of collagen and *α*-SMA, thus playing a role in the prevention and treatment of HS (134).Furthermore, melatonin promotes the apoptosis of keloid fibroblasts (KFs) through the Erk and Smad signaling pathways, inhibiting their proliferation, migration, invasion, contractility, and collagen production, thereby altering the cellular functions of KFs (135).

## 5 Clinical applications of melatonin

Dysfunction of melatonin can lead to a range of health issues, including sleep disorders, depression, and decreased immune function (136–138). This, in turn, may increase the risk of developing various conditions such as obesity, diabetes, cardiovascular diseases, and even cancer (139–141). Oral administration of melatonin follows first-order kinetics with a relatively low bioavailability of approximately 15%, which exhibits significant inter-individual variability (142, 143). Melatonin is primarily metabolized in the liver, where it is first hydroxylated to 6-hydroxymelatonin by cytochrome P450 enzymes CYP1A1 and 1A2 enzymes. The majority of this metabolite is then conjugated with sulfate, while a smaller portion is conjugated with glucuronic acid, ultimately being excreted in the urine (144). The absorption half-life is about 6 min, with serum peak concentrations reached on average 40.8 min post-administration, where the maximum concentration can reach 3550.5 pg./mL, thousands of times higher than physiological nighttime levels (125). The average elimination half-life is approximately 53.7 min (125). The low bioavailability and short half-life of melatonin pose significant challenges for its clinical application. Currently, there are melatonin-related compounds, including remelteon, being developed that can last up to 6 h (145).

The incidence of adverse reactions to melatonin is low and its effects are generally mild. Studies have shown positive effects at doses ranging from 2 to 500 mg/day, without any toxic effects (146); even doses of 100 mg/day sustained over a month have shown no substantial negative impact (147). Additionally, administration via various routes has proven to be safe (147).

While there are currently no approved melatonin or derived products for the treatment of fibrotic diseases, numerous cell and animal studies, as mentioned earlier, suggest that melatonin may have potential benefits in managing such conditions. Based on this, a few relevant clinical trials have also been initiated to clarify melatonin’s potential positive influences on fibrotic diseases. For instance, a daily supplementation of 3 mg of melatonin effectively enhanced pulmonary rehabilitation in patients with COPD (148). Similarly, in hypertensive patients, continuous use of a 1 mg/day melatonin supplement for one year effectively improved arterial stiffness (149). However, there are also reports from clinical trials that yielded contrary results. For example, administering melatonin via coronary and intravenous routes did not improve myocardial salvage index in patients with ST-segment elevation myocardial infarction (150). The timing of melatonin usage, the route of administration, and the treatment duration seem to contribute to these conflicting results. Further clinical research is needed to establish the dose–response relationship of melatonin across different diseases and to elucidate the primary mechanisms by which melatonin exerts its effects in these conditions.

## 6 Conclusion

Melatonin, as a natural neuroendocrine hormone, has good biocompatibility and safety. This review summarizes the latest advances in melatonin research and treatment for fibrosis. Increasing experimental data indicate that melatonin exerts potential antifibrotic effects in multiple tissues and organs, including the heart, lungs, liver, kidneys, and skin. Its mechanisms of action may involve reducing oxidative stress, inhibiting inflammatory responses, ECM remodeling, suppressing EMT, and regulation of apoptosis. We have identified several common molecular signaling pathways involving melatonin in fibrosis across different organs. The TGF-β pathway is crucial in nearly all types of fibrosis, as it not only has complex regulatory effects on fibrotic effector cells but also interacts with other cellular signaling pathways (97). Additionally, the NF-κB transcription factor family, as a key player in the inflammatory process, plays an important role in fibrosis across different organs (151, 152). Furthermore, the activation of the PI3K/Akt signaling pathway also contributes to the activation of various cytokines involved in fibrosis in different organs (153). Melatonin may inhibit the fibrotic process by regulating these pathways. However, the antifibrotic effects of melatonin are mostly derived from animal studies and fibrotic cell models, lacking clinical trial evidence. Despite some current research limitations, melatonin remains a promising therapeutic target with broad application prospects. Future studies should further elucidate its mechanisms of action in different organ fibrosis and develop corresponding therapeutic strategies, providing new insights and methods for treating fibrotic diseases.
